# Impact of Hybrid and Complex N-Glycans on Cell Surface Targeting of the Endogenous Chloride Cotransporter *Slc12a2*

**DOI:** 10.1155/2015/505294

**Published:** 2015-08-17

**Authors:** Richa Singh, Mohammed Mashari Almutairi, Romario Pacheco-Andrade, Mohamed Y. Mahmoud Almiahuob, Mauricio Di Fulvio

**Affiliations:** ^1^Department of Biological Sciences, Boonshoft School of Medicine, Wright State University, 3640 Colonel Glenn Highway, Dayton, OH 45435, USA; ^2^Department of Pharmacology & Toxicology, Boonshoft School of Medicine, Wright State University, 3640 Colonel Glenn Highway, 216 HSB, Dayton, OH 45435, USA

## Abstract

The Na^+^K^+^2Cl^−^ cotransporter-1 (*Slc12a2*, NKCC1) is widely distributed and involved in cell volume/ion regulation. Functional NKCC1 locates in the plasma membrane of all cells studied, particularly in the basolateral membrane of most polarized cells. Although the mechanisms involved in plasma membrane sorting of NKCC1 are poorly understood, it is assumed that N-glycosylation is necessary. Here, we characterize expression, N-glycosylation, and distribution of NKCC1 in COS7 cells. We show that ~25% of NKCC1 is complex N-glycosylated whereas the rest of it corresponds to core/high-mannose and hybrid-type N-glycosylated forms. Further, ~10% of NKCC1 reaches the plasma membrane, mostly as core/high-mannose type, whereas ~90% of NKCC1 is distributed in defined intracellular compartments. In addition, inhibition of the first step of N-glycan biosynthesis with tunicamycin decreases total and plasma membrane located NKCC1 resulting in almost undetectable cotransport function. Moreover, inhibition of N-glycan maturation with swainsonine or kifunensine increased core/hybrid-type NKCC1 expression but eliminated plasma membrane complex N-glycosylated NKCC1 and transport function. Together, these results suggest that (i) NKCC1 is delivered to the plasma membrane of COS7 cells independently of its N-glycan nature, (ii) most of NKCC1 in the plasma membrane is core/hybrid-type N-glycosylated, and (iii) the minimal proportion of complex N-glycosylated NKCC1 is functionally active.

## 1. Introduction

The Na^+^K^+^2Cl^−^ cotransporters (NKCCs) belong to the family of solute carriers* Slc12a* which comprise several homologous genes:* Slc12a1* (NKCC2),* Slc12a2* (NKCC1),* Slc12a3* (Na^+^-Cl^−^ cotransporter, NCC),* Slc12a4-7* (K^+^-Cl^−^ cotransporters, KCC1–4), and* Slc12a9* (CIP1) [1]. NKCCs are bumetanide- (BTD-) sensitive ion transporters that accumulate Cl^−^ in cells using the energy stored in the Na^+^, K^+^, and Cl^−^ chemical gradients. NKCCs play important roles in regulating the intracellular chloride concentration ([Cl^−^]_i_), a key determinant of GABAergic signaling [2], salt/fluid [3], and hormone secretion [4].

The human* SLC12A2* gene encodes at least three splice variants represented in the Reference Sequence Database of the National Center for Biotechnology Information (*RefSeq*) by accessions numbers NM_001046 (*SLC12A2 v1*, NKCC1*a*), NM_001256461 (*SLC12a2 v2*, NKCC1*b*), and NR_046207 (*SLC12A3 v3*). Except for the latter, recently annotated as a long noncoding RNA (lncRNA), NKCC1*a* and NKCC1*b* transcripts encode proteins of ~130 kDa with predicted 12-transmembrane domains and two large intracellular N- and C-termini [5]. NKCC1*b* lacks 16 C-terminal residues as a consequence of alternative splicing of exon 21 [6]. Both NKCC1*a* and NKCC1*b* exhibit similar functional properties but differ in their expression pattern [7]. With some exceptions, such as tubular cells of the thick ascending limb of Henle's loop (TALH) [8] or glucagon-secreting cells of the endocrine pancreas [9, 10], NKCC1 has been found in all mammalian cells examined so far at the protein or functional level [11]. In particular, NKCC1 localizes in the basolateral side of most epithelial cells [12–15] and polarized cell lines [16–18]. In nonpolarized cells including primary astrocytes [19] or insulin-secreting cells [10, 20], NKCC1 is found abundantly in cytoplasmic compartments. This is not extraordinary, as any transmembrane proteins including NKCCs are expected, to some extent, to be found in intracellular membrane compartments such as the endoplasmic reticulum (ER) where the transporter is synthesized and in the Golgi apparatus where complex N-glycosylation takes place [16, 21]. It is this latter step the one considered necessary for NKCC1 delivery to the plasma membrane [16]. Although N-glycosylation appears to play a role in membrane trafficking of the closely related NKCC2 [22–26], the N-glycan nature of NKCC1 and the impact of complex N-glycosylation on plasma membrane insertion of this transporter are unknown.

The objective of the present work was to determine the following: (i) the variants of NKCC1 expressed in COS7 cells, a model wherein the secretory pathway has been extensively characterized, (ii) the overall N-glycan nature of endogenous NKCC1, (iii) its cellular location, and (iv) the role of complex N-glycosylation on plasma membrane targeting and basal transport function of NKCC1. The results shown here were partially presented as posters in the annual meetings of the American Society for Biochemistry and Molecular Biology (ASBMB 2011–13) and constitute the core of RS Master's Thesis in Pharmacology and Toxicology.

## 2. Materials and Methods

### 2.1. Materials

*Pfx* DNA-polymerase, RNase-OUT, SuperScript-III reverse transcriptase, random hexamers, transfection reagents, and culture supplements including antibiotics were from Invitrogen/Life Technologies (Carlsbad, CA); dNTPs and ExoSAP-it were from Affimetrix/USB (Cleveland, OH); custom DNA primers were from Integrated DNA Technologies (Coralville, IA); the RNAeasy kit for RNA purification was from Qiagen (Valencia, CA). Human brain complementary DNA (cDNA) was obtained from Zyagen (San Diego, CA). Precasted SDS-polyacrylamide gels, running buffer, protein molecular weight (MW) markers, protease/phosphatase inhibitor cocktails, and SuperSignal West Pico Chemiluminescence kits were form Pierce (Thermo Scientific, Rockford, IL). General chemicals, cycloheximide, bumetanide, and brefeldin A were from Sigma (Saint Louis, MO). All microscopy materials were from Electron Microscopy Sciences (Hatfield, PA) and Vector Labs (Burlingame, CA). Tissue culture media and serum were from Thermo Fisher Sci. (Pittsburg, PA). Tunicamycin (TUN), kifunensine (KIF), and swainsonine (SWN) were from Cayman Chemicals (Ann Arbor, MI). DNA ladders, peptide-N-glycosidase F (PNGaseF), and endoglycosidase H (EndoH) were from New England Biolabs Inc. (Ipswich, MA).

### 2.2. Antibodies

Monoclonal antibodies against NKCC1 (T4), the lysosomal-associated membrane protein-1 (LAMP), *β*-actin, and tubulin were from Developmental Studies Hybridoma Bank (DSHB, University of Iowa). Purified mouse anti-Rab11 was from BD Transduction Laboratories (San Jose, CA). Anti-human calreticulin (CRT) and NKCC1 (ckNKCC1) antibodies raised in chicken were from Thermo Scientific (Rockford, IL). Rabbit anti-human *α*-mannosidase II (Man2) antibodies were from LifeSpan BioSciences Inc. (Seattle, WA). Conjugated secondary antibodies for immunofluorescence application or Western blotting were from Jackson Immunoresearch Laboratories Inc. (West Grove, PA).

### 2.3. Cell Culture and Stable Transfection

*Chlorocebus aethiops* (green monkey) kidney fibroblast COS7 cells (ATCC, Manassas, VA) were grown and maintained in 6-well plates (BioLite, Thermo Scientific) in high-glucose (25 mM) Dulbecco's modified Eagle medium (DMEM) supplemented with 10% fetal bovine serum (FBS), 4 mM L-glutamine, 1 mM sodium pyruvate, 100 IU/mL penicillin, 100 *μ*g/mL streptomycin, and 0.25 *μ*g/mL amphotericin B. Cells were grown in 5% CO_2_ at 37°C and media were changed every 2-3 days until ~90% confluence. To silence endogenous NKCC1 expression, early passaged COS7 cells were transfected with human lentiviral constructs encoding green fluorescent protein (GFP) and short-hairpin RNAs (shRNAs) against the fourth exon of human NKCC1 transcripts (V2LHS_93958, OpenBiosystems, Huntsville, AL). As control, we used constructs lacking shRNA sequences. Transfection was performed by using Lipofectamine 2000 and following the manufacturer's instructions. Two days after transfection, cells were washed and observed under inverted fluorescence microscope to identify GFP-expressing cells and to estimate transfection efficiency. Then, fully supplemented media containing puromycin (2.5 *μ*g/mL) were added to initiate the selection process. Western blotting with T4 antibodies was performed to screen stably silenced GFP-expressing COS7 cells.

### 2.4. RNA Extraction and RT-PCR

**Figure 1:**
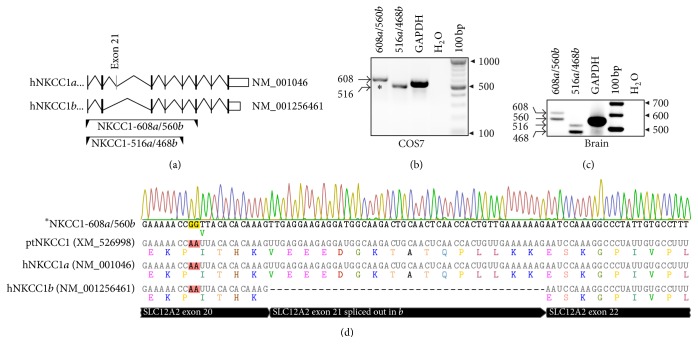
NKCC1*a* transcripts are expressed in COS7 cells. (a) Representation of the last nine exons (boxes) of the human* SLC12A2* gene. Alternative spliced exon 21 is indicated as a grey box in NKCC1*a* (NM_001046). Introns are represented with V-shaped lines. White boxes represent 3′-UTRs. Underneath, shown are the primer sets used to coamplify NKCC1*a* and NKCC1*b* mRNAs (represented as opposite arrowheads connected by a line). These primers are labeled according to the size in bp of the expected amplicons (*see text*). (b) Shown is a representative 2% agarose gel where bands of predicted sizes for NKCC1*a* are detected (608 bp and 516 bp). (c) In human brain, bands of predicted sizes for NKCC1*a* and NKCC1*b* are coamplified: 608 bp + 560 bp and 516 bp + 468 bp, respectively. As positive control, RT-PCR reactions amplified GAPDH (555 bp). As negative control, RT-PCR reactions were performed using H_2_O instead of total RNA and GAPDH-555 primer sets. (d) Representative DNA sequence chromatogram corresponding to amplicons obtained with primer set NKCC1-608*a*/560*b* encompassing exons 20, 21, and 22 in NKCC1 mRNAs. The sequences obtained from COS7 cells were aligned against chimpanzee (XM_526998) and human NKCC1 transcripts of reference. The sequence difference found in that region is highlighted.

First-strand cDNA synthesis was initiated with ~1 *μ*g of total RNA, 250 ng of random hexamers, 500 *μ*M of dNTPs, 10 mM of DTT, 40 units of RNase-OUT, and 200 units of SuperScript-III reverse transcriptase (RT) in a final volume of 20 *μ*L at 50°C for 50 min. The thermostable polymerase reaction (PCR) was subsequently performed as described in detail elsewhere [10]. Simultaneous screening of NKCC1*a* and NKCC1*b* transcripts in COS7 cells was performed following the strategy developed by Mao et al. [27] and adapted to our cell model. PCR oligonucleotide primer sets were designed using human NKCC1 transcript sequences of reference (*RefSeq* accession numbers NM_001046 and NM_001256461) as templates. The following sets of primers were used (from 5′ to 3′): NKCC1-516*a*/468*b* sense: ATG GAG TAG TGG TTA TTC GCC TAA AAG AAG, NKCC1-516*a*/468*b* antisense: TGA TAT CAG AAA AGT CTA TCC GGA ACT TGC; NKCC1-608*a*/560*b* sense: ACA TAC AAT ATG GAG TAG TGG TTA TTC GCC, NKCC1-608*a*/560*b* antisense: ATG AAG TCT GTA TGG CTC AAT GAT TTC CTC (*see* Figure 1(a), for a graphic representation of them). Control RT-PCR reactions were performed by using validated primers for human glyceraldehyde phosphate dehydrogenase (GAPDH,* RefSeq* accession number NM_002046): GAPDH-555 sense: GTG AAG GTC GGA GTC AAC GGA TTT, GAPDH-555 antisense: CAC AGT CTT CTG GGT GGC AGT GAT [28]. NKCC1 primer design and* in silico* analysis of sequences obtained were performed using the software package Geneious R7 (Biomatters, Auckland, New Zealand). The nucleotide sequence identity of DNA fragments present in PCR reaction tubes was determined after* in situ* treatment with ExoSAP-it (Affimetrix/USB, Cleveland, OH) to eliminate excess nucleotides and single stranded DNA. These noncloned but purified amplicons were sequenced by PCR using the same primer sets used to generate them (Beckman Coulter Genomics, Beverly, MA).

### 2.5. Western Blotting

COS7 cells were placed on ice and washed twice with cold phosphate buffered saline (PBS) and protein extracts were obtained by collecting cells with the help of a rubber policeman in the presence of lysis buffer [50 mM Tris-HCl pH 7.5, 1 mM EDTA pH 8.0, 1 mM EGTA pH 8.0, 0.1% (v/v) *β*-mercaptoethanol, and 1% Triton X100] supplemented with phenylmethylsulfonyl fluoride (PMSF, 25 mM) and protease/phosphatase inhibitors cocktails. Tissues lacking NKCC1 (obtained from NKCC1^KO^ mice, generated by Dr. Gary Shull, University of Cincinnati [29] and bred in our animal facility), were finely chopped on ice-cold PBS, immediately washed, and resuspended in complete lysis buffer. Samples were vigorously passed through a syringe needle while incubated for 1 h on ice. The total protein content of cell or tissue lysates was determined using Bradford's method (BioRad, Hercules, CA). Fifty *μ*g of total protein in a final volume of 20 *μ*L was mixed with an equal volume of loading buffer (8% SDS, 125 mM Tris-HCl, pH-6.8, 20% glycerol, 0.02% bromophenol blue, and 100 mM dithiothreitol) and boiled 5 minutes before loading samples onto precasted Tris-HEPES 4–20% SDS-PAGE protein gels. Samples were separated by electrophoresis for ~1 h at 100 mV. Electrophoresed proteins were then electrotransferred onto PVDF membranes (Millipore, MA) at 4°C for ~2 h. Membranes were air-dried and blocked with 5% bovine serum albumin (BSA) in 50 mM Tris, 150 mM NaCl supplemented with 0.05% Tween 20 (TBS-T) at room temperature. After blocking, membranes were cut and blotted overnight at 4°C using ckNKCC1 antibodies in 5% BSA/TBS-T. *β*-actin, tubulin, or GAPDH expression was used as internal loading controls. After blotting, membranes were washed twice with TBS-T for 5 min using gentle rocking. To develop antigen-antibody reactions, membranes were incubated with horseradish peroxidase- (HRP-) conjugated secondary antibodies directed against primary immunoglobulins for 1 h at room temperature. Membranes were then washed 5 min three times in TBS-T and developed by using chemiluminescence. Blots were visualized and images captured by using the Chemi-Doc MP Imaging system (BioRad, Hercules, CA). When required, the relative amount of NKCC1 present in immunoblots was determined by normalizing the densitometry signal to *β*-actin, tubulin, or GAPDH. Densitometry analysis was performed on digitalized blot images by using Image Lab Quantitative software (BioRad, Hercules, California) or* ImageJ* (NIH).

### 2.6. Deglycosylation

To determine the N-glycan nature of NKCC1, 50 *μ*g of cell lysates were subjected to enzymatic digestion with 50 kU of PNGaseF, an enzyme that cleaves all N-linked glycans irrespective of their nature, or EndoH, an enzyme that cleaves hybrid-type or high-mannose but not complex-type N-glycans. Digestions were performed following provider's instructions. Digested NKCC1 was immunodetected by following the immunoblotting procedures explained above.

### 2.7. Plasma Membrane Biotinylation

The extracellular surface of COS7 cells was biotinylated following published protocols [30] with the modifications published by Ortiz [31] and Nezu et al. [21]. Briefly, confluent COS7 cells growing under control conditions or in the presence of N-glycosylation inhibitors in DMEM without FBS for 16 h at 37°C were washed twice with ice-cold PBS and collected. Cells were then pelleted at 2500 ×g 5 min, resuspended in 1 mM Sulfo-NHS- (N-hydroxysuccinimide-) biotin, and incubated for 1 h at 4°C with gentle rocking. Biotinylated cells were collected by centrifugation at 2500 ×g for 4 min, washed three times with ice-cold PBS, and lysed. To separate biotinylated plasma membranes from the whole lysate mixture, 50 *μ*g of protein lysates were incubated in 150 *μ*L streptavidin-conjugated agarose resins (50% slurry plus 0.02% NaN_3_) overnight at 4°C with gentle rocking. Beads were then centrifuged at 15,000 ×g for 5 min at 4°C and washed three times with lysis buffer, three times with 1 M NaCl, and twice with PBS. Beads were then resuspended in 25 *μ*L of SDS loading buffer, denatured for 5 min at 100°C, subjected to SDS-PAGE and immunoblotted. As control of the purified membrane fraction, we used GAPDH, a cytoplasmic enzyme not present in biotinylated plasma membrane fractions [32].

### 2.8. Immunofluorescence and Coimmunolocalization Analysis

COS7 cells were grown on glass coverslips following described procedures [33]. Briefly, cells were cultured in complete DMEM until ~60–70% confluence was reached and then overnight in the absence of FBS plus or minus drugs before experiments. Cells were washed twice in PBS and immediately fixed by covering the slides in absolute methanol and incubating them for 10 min at −20°C. Cells were permeabilized in 4%* p*-formaldehyde containing 0.25% Triton X-100 for 15 min at 4°C. After washing twice with ice-cold PBS, cells were blocked against nonspecific staining by incubating them in 3% goat serum for 1 h at 4°C. Cells were then carefully washed three times with PBS at room temperature and incubated with T4 (1 : 500) alone or in combination with organelle markers: CRT (1 : 500), LAMP (1 : 1000), Rab11 (1 : 500), or Man2 (1 : 500), at 4°C overnight. The next day, immunolabeled cells were carefully washed off primary antibodies four times with ice-cold PBS and immediately incubated with fluorescently labeled secondary antibodies (Cy3- or FITC-conjugated) diluted in PBS (1 : 1000–2000) for 2 h at 4°C. After washing cells four times with PBS, the coverslips were taken from the wells and air-dried. Then, 14 *μ*L of Vectashield mounting medium containing 4′-6-diamidino-2-phenylindole (DAPI) was added to the center of the dried coverslips and immediately placed upside down on microscope slides (24 × 50 mm). Mounted slides were visualized in an Olympus Epi-Fluorescence microscope attached to a color camera using 60x oil objective and FITC/Cy3/DAPI fluorescence filters. Photomicrographs were obtained using Diagnostics Instrument Spot 6 digital camera and MetaVue software (Molecular Devices, Sunnyvale, CA). Coimmunolabeling analysis was based on relative semiquantitation of overlapping pixels observed in high-resolution digital images taken using different filters/channels and superimposed (overlays). This estimation was performed* in silico* and expressed as % overlap. We considered colocalization when pixels of different channels shared the same bit area. All pixels were analyzed based on their intensity as interpreted in the algorithms used by the software suite* Metamorph* (Molecular Devices, Sunnyvale, CA) with minimal user interference. In general, selected areas of the cells were measured for specific florescence signal and the data obtained was plotted using* Graphpad Prism* v5 (San Diego, CA). To estimate colocalization, heat-maps were produced by counting pixels for each gray value on 8-bit transformed overlay pictures using the heat-map plugin of* ImageJ* (NIH).

### 2.9. Chloride Uptake

COS7 cells were seeded onto 6-well plastic plates and grown until ~80% confluence. Cells were washed and preincubated in Cl^−^-free isotonic solution containing (in mM) 20 Tris-HEPES (pH 7.4), 130 Na^+^ gluconate, 5 K^+^ gluconate, 2 Ca^2+^ gluconate, 1 MgSO_4_, and 10 glucose, for 60 min at room temperature. After this preincubation period, media were aspirated and replaced by an isotonic solution where gluconate salts were replaced in a mol-to-mol basis with the respective Cl^−^ salts. Cl^−^ uptake was terminated with three washes in ice-cold Cl^−^-free isotonic solution and the ionic content of cells released by incubating them in 0.25 N NaOH at RT for 30 min. After neutralization with glacial acetic acid, aliquots were accurately taken for protein determination and measurement of Cl^−^ with calibrated ion-selective electrodes (Thermo Sci-Orion). The relationship between concentration of Cl^−^ and the response of the Cl^−^ electrode in mV at 25°C is described by the Nernst equation: *E* (mV) = *k* − 59.16 × log⁡[Cl^−^]. Electrode responses were converted to [Cl^−^] in standard curves *E versus *log⁡[Cl^−^]. Then, net Cl^−^ uptake was calculated and expressed as nmol of Cl^−^ per *μ*g of total protein. Bumetanide- (BTD-) sensitive Cl^−^ uptake (NKCC-mediated) was defined as the difference between Cl^−^ accumulated in 5 minutes and that obtained in the presence of 10 *μ*M BTD.

*Note*. The response of the electrode to variable concentrations of BTD was negligible.

### 2.10. Statistical Analysis

Analysis of multiple group differences was performed using one-way analysis of variance (ANOVA) followed by Student-Newman-Keuls' test. A *p* value less than 0.05 was used as the criteria of statistical significance.

## 3. Results

### 3.1. COS7 Cells Express NKCC1*a*

To identify NKCC1 splice variants expressed in COS7 cells, we used RT-PCR and two sets of primers encompassing the junction between exons 20, 21 [48 base pairs (bp)], and 22. These sets of primers named NKCC1-608a/560b and NKCC1-516a/468b are predicted to coamplify NKCC1*a* and NKCC1*b* mRNAs as bands of 608 or 516 bp and 560 or 468 bp, respectively. A representation of these primers relative to the relevant region of human NKCC1 transcripts is shown in Figure 1(a). As shown in Figures 1(b) and 1(c), NKCC1*a* transcripts bands of 608 and 516 bp are detected in COS7 cells whereas bands of 560 and 468 bp corresponding to NKCC1*b* were not coamplified suggesting that NKCC1*a* mRNAs are expressed in these cells. The undetectable levels of NKCC1*b* transcripts are not related to poor primer design; NKCC1*a* and NKCC1*b* mRNAs are codetected as bands of 608, 560, 516, and 468 bp in cDNA samples obtained from human brain, an abundant source of NKCC1*a* and NKCC1*b* transcripts [7]. To further corroborate the presence of NKCC1*a* in COS7 cells, the whole RT-PCR reaction carried out with primer set NKCC1-608a/560b (asterisk in Figure 1(b)) was purified and sequenced. As shown in Figure 1(d), the nucleotide sequence obtained identified exon 21 of NKCC1*a* but not the junction of exons 20 and 22 demonstrating that NKCC1-608a/560b amplified NKCC1*a* mRNAs from COS7 cells. Of note, we did not find evidence of* Slc12a2 v3* lncRNAs in human brain or COS7 cells (*results not shown*).

**Figure 2:**
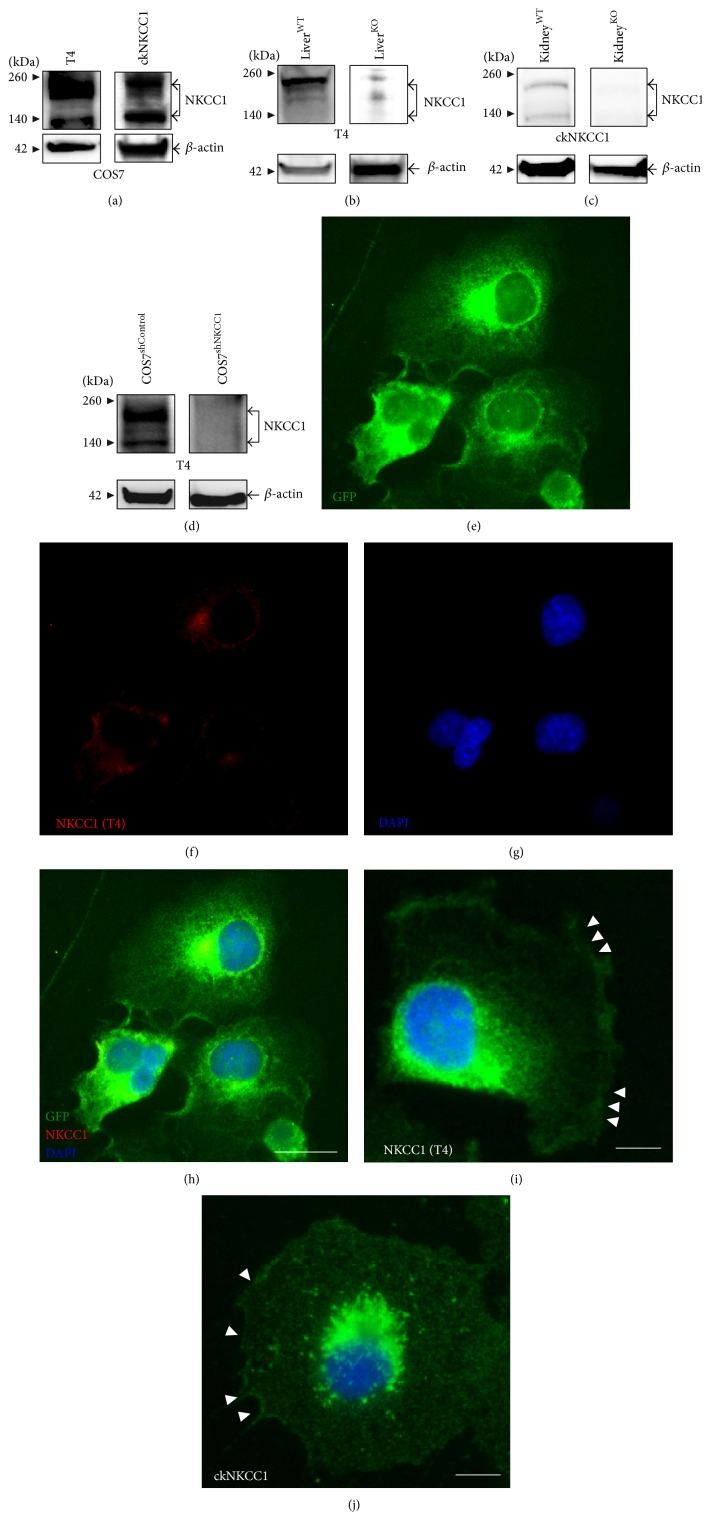
Validation of NKCC1 antibodies in COS7 cells. (a) Representative immunoblot of protein lysates obtained from COS7 cells and developed by using T4 or ckNKCC1. Shown is NKCC1 protein expression detected as two major bands, one of ~130 kDa and another of ~170 kDa. ((b)-(c)) Representative immunoblots of liver (b) or kidney (c) tissue extracts from WT or NKCC1^KO^ mice, developed by using T4 (b) or ckNKCC1 (c). Shown are bands of predicted MWs corresponding to NKCC1 only in WT tissues. (d) Representative immunoblots of COS7^shControl^ and COS7^shNKCC1^ protein extracts developed by using T4. Shown are bands of predicted MWs corresponding to NKCC1 only in COS7^shControl^ cells. ((e)–(h)) Representative images of COS7^shNKCC1^ cells stably expressing shRNAs against NKCC1 and GFP as a reporter (e) immunolabeled by using T4 coupled to Cy3-conjugated secondary antibodies (f). Cell nuclei were counterstained with DAPI (g) and the pictures superimposed (h). ((i)-(j)) Immunofluorescence of COS7 cells using T4 (i) or ckNKCC1 (j) coupled to FITC-conjugated secondary antibodies. Arrowheads indicate NKCC1-immunoreactivity at/near the edge of cells. Pictures in (e)–(h) and (i)-(j) were taken at 400x and 600x, respectively. Bar represents 10 *μ*m.

To confirm NKCC1 expression at the protein level in COS7 cells, we performed immunoblotting experiments using two different antibodies directed against human NKCC1. These are T4, a thoroughly characterized, widely used, and validated mouse monoclonal antibody [34] and a recently available polyclonal antibody raised in chicken (ckNKCC1). As shown in Figure 2(a), T4 and ckNKCC1 detect NKCC1 in COS7 cells as two major bands, one of ~130 kDa and another of ~170 kDa, considered the core/high-mannose and complex N-glycosylated forms of the transporter, respectively [16, 35–39]. To minimize the possibility of cross-reaction with unrelated proteins, we tested ckNKCC1 in blots of protein extracts obtained from NKCC1^KO^ tissues. Figures 2(b) and 2(c) show that T4 and ckNKCC1 do not detect proteins in liver or kidney of NKCC1^KO^ mice, respectively. It is important to note that the kidney is the most abundant source of NKCC2 [3], the only known cross-target of T4 [40]. To validate T4 in our cell model, this antibody was tested in COS7 cells depleted of endogenous NKCC1 (COS7^shNKCC1^). As control, COS7 cells were stably transfected with constructs lacking shRNAs (COS7^shControl^).  Figure 2(d) shows that T4 does not detect NKCC1 in protein extracts from COS7^shNKCC1^, a finding that correlates at the cellular level. Indeed, T4 was unable to detect immunoreactive NKCC1 in fixed COS7^shNKCC1^ cells (Figures 2(e)–2(h)) suggesting that the antibody is specific of NKCC1 in COS7 cells under our experimental conditions. To further support the concept that T4 and ckNKCC1 produce comparable results, normal COS7 cells were immunolabeled in parallel with each antibody. As shown in Figures 2(i) and 2(j), T4 and ckNKCC1 detect NKCC1 distributed throughout the cells in a reticular, granular, or vesicular pattern and fairly concentrated toward one of the poles of the nucleus as well as in the perinuclear region and near or at the plasma membrane. Taken together, these results suggest that core/high-mannose/hybrid/complex N-glycosylated NKCC1*a* is the sole NKCC1 variant expressed in COS7 cells and that ckNKCC1 and T4 antibodies may be used to determine NKCC1 protein expression patterns at the molecular and cellular levels in our cell model.

### 3.2. A Small Proportion of Endogenous NKCC1 Is Complex N-Glycosylated

**Figure 3:**
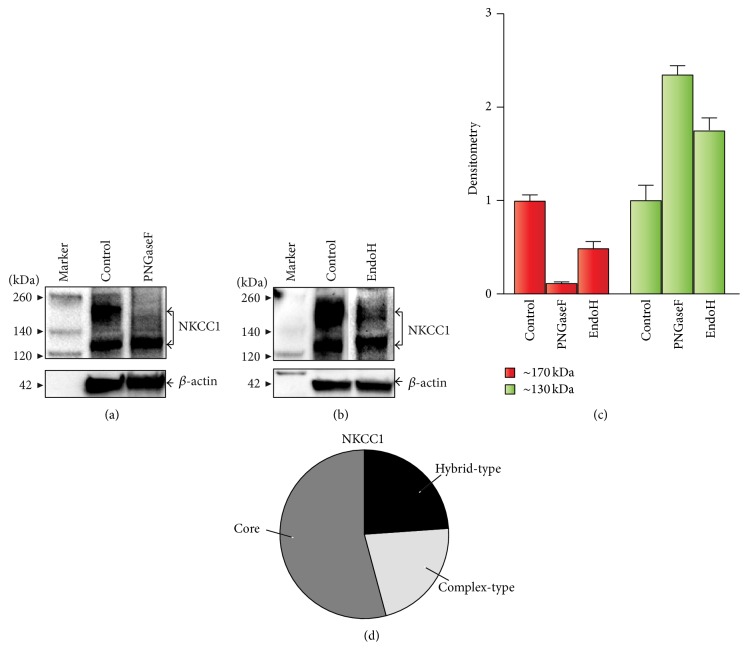
NKCC1 is expressed in COS7 cells as a core/high-mannose, hybrid- and complex-type N-glycosylated protein. ((a)-(b)) Representative immunoblots of protein extracts from COS7 cells grown under basal conditions. Untreated protein extracts (control) or enzymatically digested with PNGaseF (a) or EndoH (b) were immunoblotted and developed using ckNKCC1 coupled to HRP-conjugated secondary antibodies. As loading control, membranes were probed with antibodies against *β*-actin. (c) Densitometry analysis of immunoblots of proteins control or treated with PNGaseF or EndoH. Shown are the estimated levels of the two main bands of NKCC1, that is, ~170 kDa (red bars) and ~130 kDa (green bars), before and after enzymatic deglycosylation. Data are expressed as means ± SEM (*n* = 5). (d) Pie-chart representing the approximate relative contribution of core/high-mannose, hybrid- and complex-type N-glycans to total endogenous NKCC1 in COS7 cells. Results are calculated based on the percent of increase in the densitometry signal of bands of ~130 kDa after enzymatic treatment.

Enzymatic deglycosylation coupled to immunoblotting was used to determine the basal N-glycosylation state and global N-glycan nature of endogenous NKCC1 expressed in COS7 cells. To this end, protein lysates were treated with PNGaseF or EndoH, enzymes that cleave all N-linked glycans or high-mannose/hybrid-type, respectively. As shown in Figure 3(a), treatment with PNGaseF resulted in a significant, nearly complete elimination of the ~170 kDa band and a parallel ~2-fold increase in core NKCC1 levels (~130 kDa). These results suggest that at least half of total NKCC1 expressed in COS7 cells is core/high-mannose N-glycosylated. Further, as shown in Figure 3(b), treatment of COS7 cells protein extracts with EndoH resulted in ~50% decrease of the ~170 kDa bands of NKCC1 indicating that half of the total N-glycan composition of NKCC1 is of the core/high-mannose/hybrid-type. To visualize these changes, immunoblots were subjected to densitometry analysis. Figures 3(c) and 3(d) show the estimated relative contribution of core and hybrid- and complex-type N-glycans to the total pool of endogenous NKCC1, a parameter calculated as percentage densitometry increase in the bands of ~130 kDa after deglycosylation. To interpret these results, we applied the following reasoning: if the % densitometry signal of ~130 kDa bands increased twice after PNGaseF treatment, then 50% of total NKCC1 is N-glycosylated. Similarly, if the % densitometry signal of ~170 kDa bands after EndoH treatment was half, then ~25% of total NKCC1 is hybrid-type N-glycosylated. Therefore, our results suggest that ~25% of total NKCC1 expressed in COS7 cells is complex N-glycosylated.

### 3.3. Core/High-Mannose, Hybrid, and Complex N-Glycosylated NKCC1 Reach the Plasma Membrane

**Figure 4:**
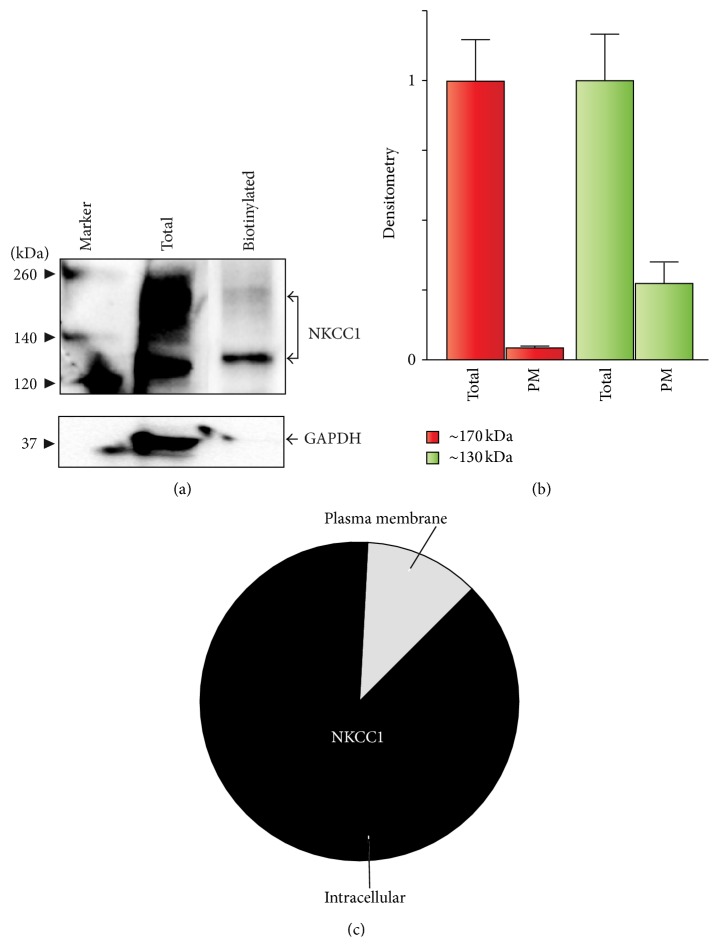
A small proportion of total NKCC1 locates in the plasma membrane. (a) Representative NKCC1 immunoblot of equal amounts of total protein extracts and plasma membrane biotinylated fractions obtained from COS7 cells growing under normal conditions. Shown are bands of expected sizes of NKCC1, that is, ~170 kDa and ~130 kDa, in total cellular extracts and in biotinylated plasma membrane fractions. Protein expression of cytosolic GAPDH was used as a loading control for total protein lysates and to assess the purity of biotinylated plasma membrane fractions. (b) Densitometry analysis of immunoblots showing estimated levels of the two main immunobands of NKCC1: core/high-mannose ~130 kDa (green bars) and complex/hybrid ~170 kDa (red bars) in total lysates or biotinylated plasma membrane fractions. Data is expressed as the mean ± SEM (*n* = 3). (c) Pie-chart representation of the estimated, relative contribution of total NKCC1 to the plasma membrane.

It is generally accepted that complex N-glycosylation of NKCCs is necessary for plasma membrane insertion [16, 21, 23–26]. Therefore, the results presented in Figure 3 predict that at least a quarter of total NKCC1 locates in the plasma membrane. To corroborate this prediction, the immunological presence of NKCC1 was determined in total protein extracts and in biotinylated plasma membrane fractions purified from COS7 cells growing under normal conditions. As shown in Figure 4(a), a small relative proportion of NKCC1 bands of ~130 kDa and ~170 kDa were found in the biotinylated fraction indicating that core/high-mannose and hybrid/complex-type N-glycosylated NKCC1 reach the plasma membrane. Of note, the facts that (i) intact cells were biotinylated, collected, lysed, and then affinity-purified with streptavidin-coated agarose beads and (ii) cytosolic GAPDH is minimally detected in blots of purified biotinylated fractions all indicate that NKCC1 of ~130 kDa represents plasma membrane located NKCC1 rather than a contamination from potentially biotinylated intracellular compartments. To estimate the relative contribution of these N-glycans to plasma membrane NKCC1, we performed densitometry analysis of immunoblots loaded with equivalent amounts of protein. As shown in Figures 4(b) and 4(c), ~10% of total NKCC1 is found in the plasma membrane, mostly corresponding to the core/high-mannose or hybrid forms of the transporter. Therefore, these results suggest that the overall N-glycan nature of NKCC1 does not determine its plasma membrane location.

### 3.4. Endogenous NKCC1 Locates in ER, Rab11-Positive Compartments, and Medial-Golgi Cisternae

**Figure 5:**
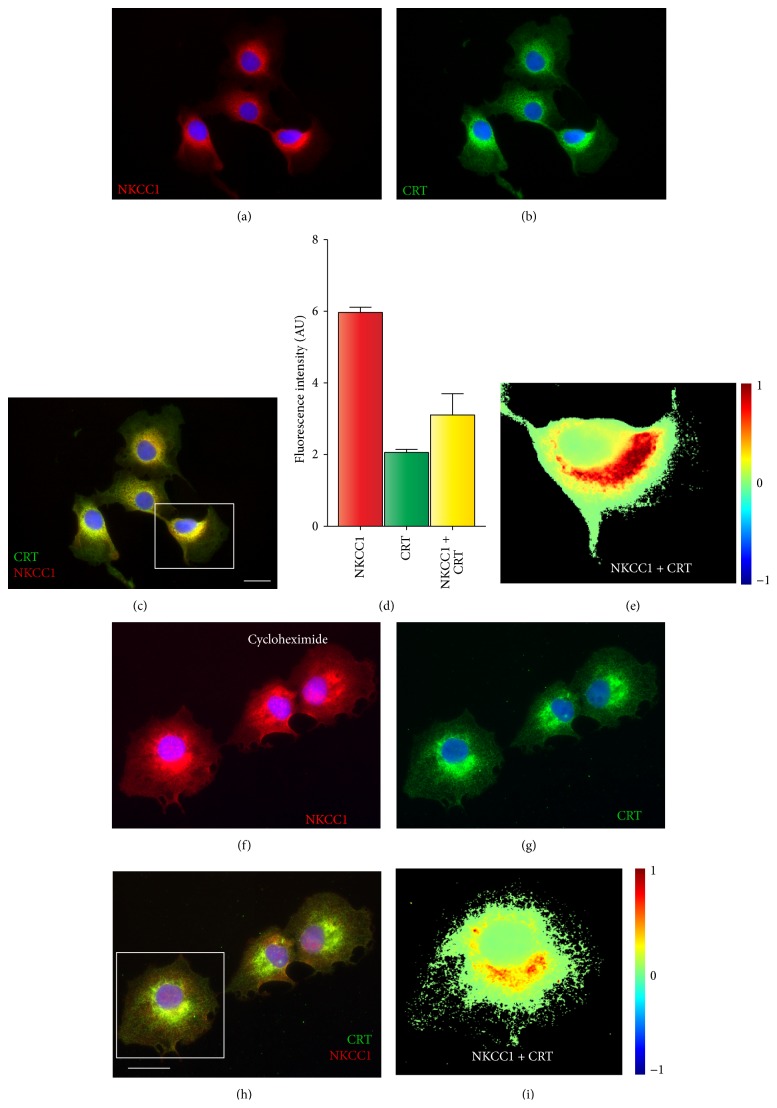
NKCC1 localizes in the ER of COS7 cells. ((a)-(b)) Representative image of COS7 cells immunolabeled against NKCC1 using T4 (a) or calreticulin (CRT, (b)) antibodies and developed using fluorescently labeled secondary antibodies: Cy3 (NKCC1, red) and FITC (CRT, green). (c) Overlay of (a) and (b). Pictures were superimposed to obtain an image where colocalization could be digitally estimated as yellow pixels (red + green = yellow). Scale bar represents 10 *μ*m. (d) Semiquantitation of red, green, and yellow pixels corresponding to NKCC1, CRT, and NKCC1 + CRT, respectively. Shown are the results obtained from at least 10 cells and represented as mean fluorescence intensity in arbitrary units ± SEM. (e) Colocalization heat-map of the squared cell in (c) computed by using NIH* ImageJ*. ((f)-(g)) Shown are representative images of COS7 cells grown in the presence of cycloheximide (1 *μ*g/mL) to acutely inhibit protein synthesis and deplete endogenous NKCC1 from the ER. The presence of NKCC1 (f) or CRT (g) was codetected by using the relevant primary antibodies and developed using secondary antibodies labeled with Cy3 (red) or FITC (green) fluorophores. (h) Superimposition of (f) and (g) images to estimate colocalization as heat-maps, as shown in (i).

If half of endogenous NKCC1 is core/high-mannose N-glycosylated (Figure 3), then the transporter locates in the endoplasmic reticulum (ER) in a similar proportion. To determine the immunolocalization pattern of NKCC1 in COS7 cells, we used immunofluorescence microscopy coupled to antibodies directed against calreticulin (CRT), a validated ER marker [41]. As shown in Figures 5(a)–5(c), immunoreactive NKCC1 locates in CRT-positive compartments, confirming ER location of the transporter. To estimate the extent of NKCC1 ER localization, the spatial overlap between NKCC1 (*red*) and CRT (*green*) signals in immunofluorescence images was quantified* in silico*. As shown in Figure 5(d), ~50% of total NKCC1-related signal overlaps with that of CRT, a proportion that correlates the EndoH-sensitive NKCC1 signal obtained in immunoblots (*see* Figure 3(b)). From the immunofluorescence data presented in Figures 5(a)–5(c), it is evident that NKCC1 also locates in CRT-negative compartments. To visualize the extrareticular component of NKCC1 localization in COS7 cells, ongoing NKCC1 biosynthesis in the ER was inhibited with a 2 h pulse of cycloheximide (CHX); then, cells were fixed and immunolabeled against CRT. As shown in Figures 5(f)–5(i), inhibition of protein synthesis depletes NKCC1 from CRT-positive compartments evidencing a vesicular, post-ER cytoplasmic pattern of NKCC1 expression in these cells.

**Figure 6:**
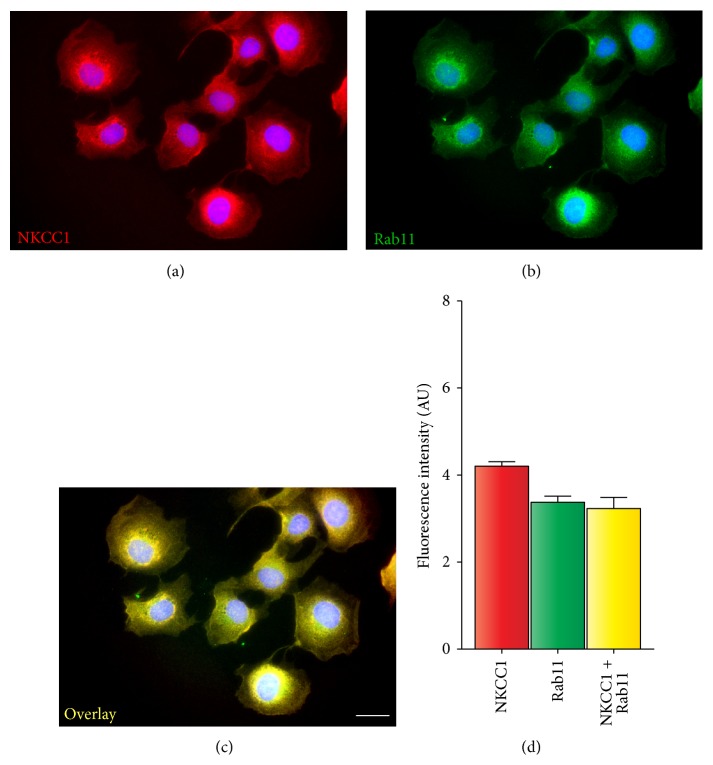
NKCC1 localizes in Rab11-positive compartments in COS7 cells. ((a)-(b)) Shown are representative images of COS7 cells expressing immunoreactive NKCC1 (a) or Rab11 (b). (c) (a) + (b) images superimposed to obtain an overlay image where colocalization could be estimated as yellow pixels (red + green). The scale bar represents 10 *μ*m. (d) Semiquantitation of red (NKCC1), green (Rab11), and yellow (NKCC1 + Rab11) pixels. Shown are results obtained from at least 10 cells and represented as mean fluorescence intensity in arbitrary units ± SEM.

**Figure 7:**
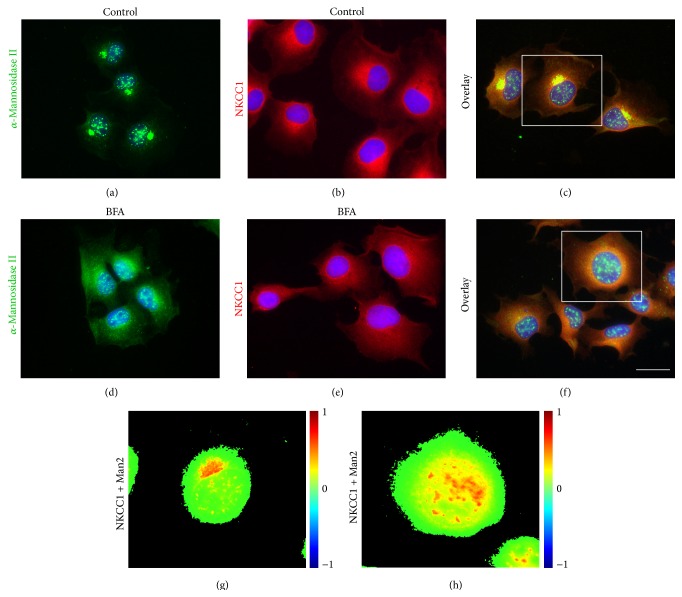
NKCC1 localizes in* cis*/medial-Golgi cisternae. ((a)–(c)) Shown are representative images of COS7 cells grown under control conditions or treated for 16 h with 1 *μ*g/mL of brefeldin A (BFA, (d)–(f)). Immunoreactive *α*-mannosidase II (Man2, (a) and (d)) and NKCC1 ((b) and (e)) were detected by using primary antibodies directed against these proteins and developed using secondary antibodies labeled with FITC (green) and Cy3 (red), respectively. ((c) and (f)) Shown are superimposed images to obtain an overlay representing colocalization of NKCC1 and Man2. The scale bar represents 10 *μ*m. ((g)-(h)) Heat-maps of the squared cells in (c) and (f), respectively, computed by using NIH* ImageJ*.

To identify these additional CRT-negative intracellular compartments, NKCC1 was coimmunolabeled with Rab11, a small GTPase considered a steady-state marker of* trans*-Golgi network, secretory granules, constitutive exocytic vesicles, and recycling/sorting endosomes [42, 43]. As shown in Figures 6(a)–6(d), ~90% of endogenous NKCC1 locates in Rab11-positive compartments consistent with the notion that NKCC1 is largely confined to the intracellular space. Moreover, these observations indicate that NKCC1 may constitutively traffic between these Rab11-positive compartments including recycling endosomes, ER and Golgi. Therefore, we tested whether NKCC1 is present in Golgi structures. To this end, we used Man2, a marker of* cis*/medial-Golgi cisternae [44, 45]. As shown in Figures 7(a)–7(c), NKCC1 appears concentrated in structures immunolabeled with Man2 demonstrating that NKCC1 locates in the* cis*/medial-Golgi. To add support to this conclusion, Golgi cisternae were collapsed into the ER by using brefeldin A (BFA) [46, 47]. As shown in Figures 7(d)–7(f), BFA treatment resulted in redistribution of NKCC1 and Man2 to the perinuclear region of the cells. Therefore, altogether, these results indicate that the intracellular location of endogenous core/high-mannose and hybrid/complex-type N-glycosylated NKCC1 in ER, Rab11-positive vesicles, and* cis*/medial-Golgi cisternae reflects its biosynthetic pathway.

### 3.5. Inhibition of the First Step in N-Glycan Biosynthesis Impairs NKCC1 Expression

**Figure 8:**
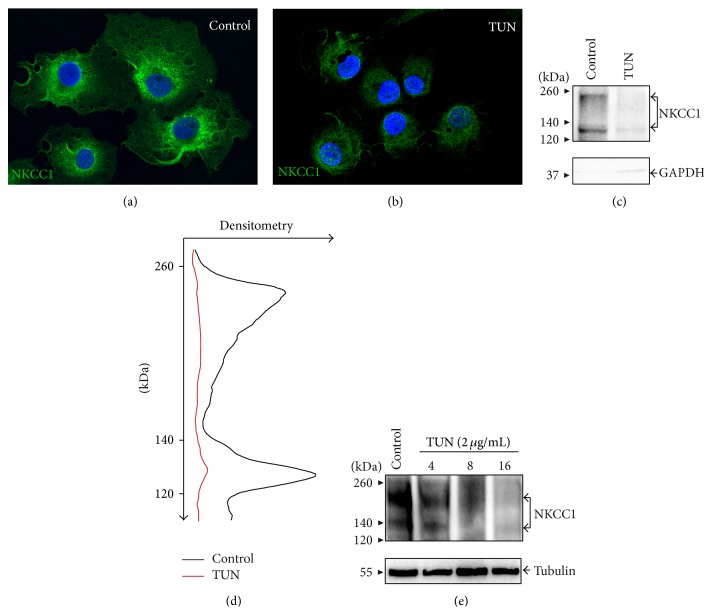
The first step of N-glycan biosynthesis is required for NKCC1 protein expression. ((a)-(b)) Shown are representative immunofluorescence microscopy images of COS7 cells grown under control conditions (a) or treated for 16 h with 2 *μ*g/mL tunicamycin (TUN, (b)). NKCC1 immunolocalization was analyzed with T4 and FITC-labeled secondary antibodies (green). (c) Representative immunoblot with ckNKCC1 demonstrating expression of NKCC1 in the biotinylated plasma membrane fraction purified from COS7 cells control or treated with TUN. Protein expression of cytosolic GAPDH was used to assess the purity of biotinylated plasma membrane fractions. (d) Densitometry scanning of the immunoblot in (c) representing the extent to which TUN (red trace) decreases plasma membrane located NKCC1 (black trace). (e) Representative immunoblot showing the expression pattern of total NKCC1 in COS7 cells in response to TUN 2 *μ*g/mL during the indicated periods of time. As loading control, immunoblots were probed against tubulin.

To study the role of N-glycosylation on endogenous NKCC1 trafficking and localization, we first treated cells with tunicamycin (TUN, 2 *μ*g/mL for 16 h), an inhibitor of the first step of N-glycan biosynthesis. As shown in Figures 8(a) and 8(b), TUN significantly decreases total immunoreactive NKCC1 protein expression levels consistent with the notion that core N-glycosylation of proteins plays a key role in protein stability and quality control in the ER [48–50]. To correlate these results at the plasma membrane level, TUN-treated cells were biotinylated and plasma membrane fractions were purified and subjected to immunoblotting. As shown in Figures 8(c) and 8(d), TUN decreased expression of plasma membrane located NKCC1. Since TUN also decreased total expression levels of NKCC1, as seen in Figures 8(d) and 8(e), these results suggest that the extent of plasma membrane located NKCC1 reflects its biosynthesis levels rather than its N-glycosylation state.

### 3.6. Complex N-Glycosylation Is Not Required for Plasma Membrane Targeting of NKCC1 but for Its Cotransport Function

**Figure 9:**
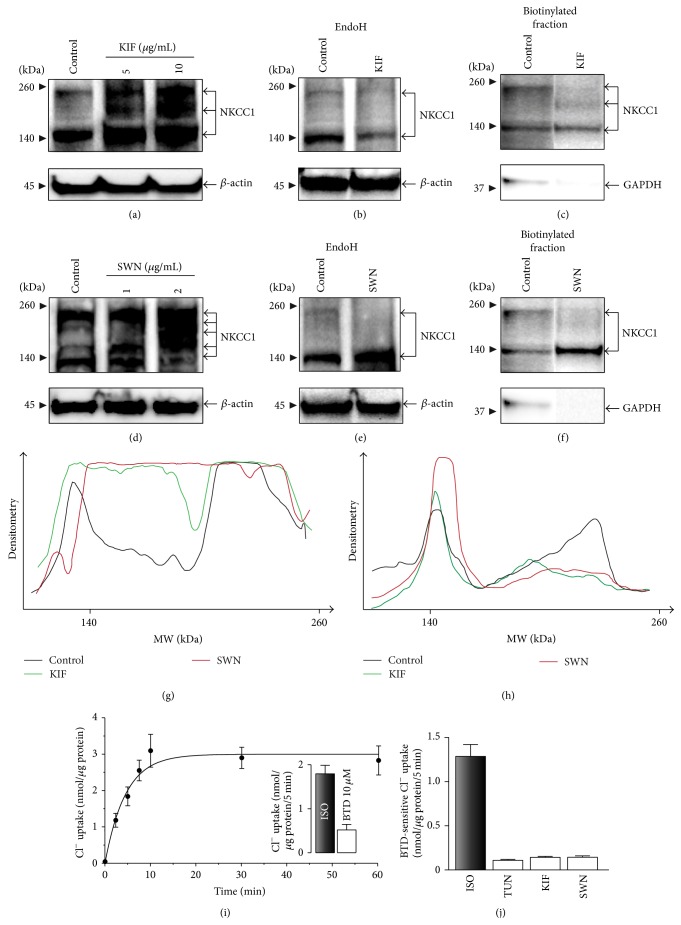
Inhibition of N-glycan processing or complex N-glycosylation does not preclude NKCC1 plasma membrane localization but impairs its function. ((a) and (d)) Shown are representative immunoblots experiments showing total endogenous expression levels of NKCC1*a* in COS7 cells incubated with (DMSO, control) 5–10 *μ*g/mL of kifunensine (KIF, (a)) or 1-2 *μ*g/mL swainsonine (SWN, (d)) for 16 h. Note that the expected bands of NKCC1*a*, that is, ~130 kDa and ~170 kDa, are detected in control and treated cells whereas additional ckNKCC1-immunoreactive bands centered at ~150 kDa are observed only in protein extracts from KIF/SWN-treated cells. As loading control, immunoblots were developed using antibodies directed against *β*-actin. ((b) and (e)) Shown are representative immunoblots demonstrating expression of EndoH-sensitive hybrid-type N-glycosylated NKCC1*a* in total protein extracts of COS7 cells treated for 16 hs with KIF (10 *μ*g/mL) or SWN (2 *μ*g/mL). Note the absence of immunoreactive bands corresponding to NKCC1*a* ~170 kDa in cell extracts obtained from KIF/SWN-treated cells. ((c) and (f)) Shown are representative immunoblots demonstrating expression of NKCC1*a* in biotinylated plasma membrane fractions of COS7 cells grown under control conditions or treated for 16 h with KIF or SWN. Protein expression of the cytosolic GAPDH was used to assess the purity of the plasma membrane fractions. (g) Densitometry scanning representing the total cellular N-glycan types of NKCC1 in COS7 cells control or treated for 16 h with KIF (10 *μ*g/mL) or SWN (2 *μ*g/mL). (h) Representative densitometry scanning of NKCC1 immunoblots of plasma membrane fractions obtained from COS7 cells control or treated for 16 h with KIF (10 *μ*g/mL) or SWN (2 *μ*g/mL). (i) Cl^−^ uptake into COS7 cells depleted of endogenous Cl^−^ as a function of time (0–60 min). Uptake is represented as nmol/*μ*g protein [mean ± SEM (*n* = 6)].* Inset:* mean Cl^−^ uptake [nmol/*μ*g protein/5 min ± SEM (*n* = 5)] obtained under isotonic conditions (ISO, black bar) or in the presence of BTD 10 *μ*M (ISO + BTD, white bar). (j) Impact of TUN (2 *μ*g/mL), KIF (10 *μ*g/mL), or SWN (1 *μ*g/mL) treatment (16 hs) on the BTD-sensitive component of Cl^−^ uptake into COS7 cells. Results are expressed as nmol/*μ*g protein/5 min ± SEM (*n* = 3).

Like in the case of NKCC2 engineered to lack consensus N-glycosylation sites [21–26], precluding N-glycosylation with TUN impairs N-glycan maturation and therefore the role of the latter in plasma membrane targeting cannot be addressed with these kinds of experiments. To determine the role of N-glycan maturation in plasma membrane targeting of endogenous NKCC1, cells were treated with kifunensine (KIF, 5–10 *μ*g/mL), an inhibitor of ER-located mannosidase-I and complex N-glycosylation [51–53]. As shown in Figure 9(a), KIF treatment results in increased expression levels of core/high-mannose and hybrid-type N-glycosylated NKCC1 (Figure 9(g), green trace), as expected for inhibition of ER-mannosidase-I [52, 54]. To confirm the hybrid N-glycan nature of KIF-treated NKCC1, protein extracts obtained from KIF-treated COS7 cells were subjected to enzymatic digestion with EndoH. As shown in Figure 9(b), EndoH treatment results in a nearly complete elimination of NKCC1 bands heavier than ~130 kDa, indicating that KIF impairs NKCC1 complex N-glycosylation. At the plasma membrane level, KIF treatment did not impact core/high-mannose NKCC1 (~130 kDa) expression levels but resulted in almost undetectable complex N-glycosylated NKCC1 and a parallel increase in the hybrid-type form (Figures 9(c) and 9(h), green trace). Therefore, these results suggest that inhibition of complex-type N-glycosylation does not impact plasma membrane location of NKCC1. To further substantiate these findings, expression of the transporter was determined in COS7 cells treated with swainsonine (SWN, 1-2 *μ*g/mL), an inhibitor of medial-Golgi Man2, the enzyme involved in the first biosynthetic step of complex N-glycosylation [55, 56]. As shown in Figure 9(d), SWN increases precursor core/high-mannose and hybrid-type N-glycosylated NKCC1 expression (Figure 9(g), red trace), as expected. Further, as shown in Figure 9(e), EndoH digestion of SWN-treated samples resulted in complete elimination of NKCC1 bands higher than ~130 kDa, but not in control samples where bands of ~170 kDa complex N-glycosylated, EndoH-resistant NKCC1 proteins are observed suggesting complete inhibition of complex N-glycosylation of NKCC1 by SWN. At the plasma membrane level, SWN also eliminated complex N-glycosylated NKCC1 (Figures 9(f) and 9(h)). However, SWN treatment increased the levels of core/high-mannose and hybrid-type N-glycosylated NKCC1 paralleling the increase in their total expression levels. Together, these results confirm that plasma membrane targeting of NKCC1 is independent of its N-glycosylation state.

To verify that plasma membrane targeted NKCC1 either core/high-mannose, hybrid-type, or complex N-glycosylated is functional, we first determined total NKCC activity in COS7 cells depleted of endogenous Cl^−^ as net Cl^−^ uptake as a function of time. As shown in Figure 9(i), Cl^−^ uptake into COS7 cells reaches a plateau after ~10 minutes at room temperature and a final extracellular [Cl^−^]_o_ of 139 mM. To determine the NKCC-dependent component of Cl^−^ uptake, net Cl^−^ transport into Cl^−^-depleted cells was determined in the presence of 10 *μ*M bumetanide (BTD). This was assessed at saturating concentrations of Cl^−^ (139 mM), 5 minutes after incubation of Cl^−^-depleted cells in isotonic (ISO) media. Under these conditions, the BTD-sensitive component of total Cl^−^ uptake reached ~65% (*inset*, Figure 9(i)), suggesting that the small proportion of total NKCC1 expressed in the plasma membrane, either core/high-mannose or hybrid- or complex-type, performs functional work. To determine the role of global or complex N-glycosylation on the cotransport function of plasma membrane located NKCC1, cells were incubated in the presence of TUN, KIF, or SWN under conditions comparable to those used for protein expression analyses. As shown in Figure 9(j), total BTD-sensitive Cl^−^ uptake is almost completely eliminated in COS7 cells treated with TUN, as expected for a drug that heavily reduces total NKCC1 expression levels (*see* Figure 8). However, treatment of COS7 cells with KIF or SWN which only eliminated complex N-glycosylated NKCC1 at the plasma membrane blocked BTD-sensitive Cl^−^ uptake, suggesting that complex N-glycosylation is required for the transporter to function and that core/high-mannose or hybrid-type NKCC1 cannot transport Cl^−^ despite their plasma membrane location.

## 4. Discussion

NKCC1 is ubiquitously expressed [57]. However, which NKCC1 splice variants are present in a particular cell model or tissue is frequently unknown. By using RT-PCR, we have provided evidence of NKCC1*a* expression in COS7 cells (Figures 1(b) and 1(c)). A single RT-PCR amplicon was obtained from these cells by using a validated RT-PCR strategy designed to simultaneously coamplify NKCC1*a* and NKCC1*b* transcripts [27] (Figure 1(c)). In addition, undetectable levels of NKCC1*b* transcripts in our cell model are supported by the sole presence of nucleotide sequences corresponding to exon 21 in sequencing chromatograms (Figure 1(d)). Therefore, our results suggest that NKCC1*a* represents most if not all of the total pool of NKCC1 in COS7 cells. Expression of NKCC1 at the protein level in COS7 cells was initially studied using two different antibodies directed against the transporter, that is, T4 and ckNKCC1 (Figure 2). This was performed to validate NKCC1 antibodies in our cell model based on the findings that T4 detects NKCC2, at least when abundantly expressed, as it is the case of the kidney or when forcedly overexpressed in cell models [40, 58]. Notably, COS7 cells express very low levels of NKCC2 (*data not shown*). Nevertheless, T4 did not detect NKCC2 in COS7 cells silenced of endogenous NKCC1 or in NKCC1^KO^ tissues indicating that the ability of T4 to cross-react with NKCC2, or any other protein, may depend on its expression levels. Similarly, ckNKCC1 did not detect proteins in immunoblots of kidney extracts obtained from NKCC1^KO^ mice demonstrating that this antibody does not cross-react with NKCC2 (Figure 2(c)). In addition, our results demonstrate that both T4 and ckNKCC1 detect similar patterns of NKCC1 expression in all protein samples tested, that is, two bands of ~130 and ~170 kDa, and in fixed cells, that is, intracellular and plasma membrane located NKCC1 (Figures 2(g) and 2(h)). Therefore, these results validate the use of these antibodies to study expression, N-glycan composition of NKCC1, and its localization in COS7 cells, the main interests of the present study.

Our results suggest that NKCC1 is expressed in COS7 cells as an N-glycosylated protein (Figures 2–5 and 9), in agreement with many reports [21, 35–39, 59–63]. However, these reports concluded that NKCC1 is a complex N-glycosylated protein based on digestion experiments using PNGaseF, an amidase that cannot distinguish core/hybrid from complex N-glycans. Our experiments determined the N-glycan nature of NKCC1 in COS7 cells (Figure 3). PNGaseF treatment resulted in an almost complete elimination of the ~170 kDa N-glycan NKCC1 bands, as expected, but also resulted in a concomitant ~2-fold increase in the band of ~130 kDa. The most parsimonious interpretation of these results is that ~50% of total NKCC1 is hybrid/complex-type N-glycan (Figures 3(c) and 3(d)). In addition, the results after EndoH treatment suggest that approximately half of the total N-glycan load of NKCC1 corresponds to the high-mannose/hybrid-type and that only ~25% is of the complex nature that is resistant to the action of EndoH (Figures 3(c) and 3(d)). These data are consistent with the interpretation that endogenous NKCC1 is expressed in COS7 cells as core/high-mannose and hybrid/complex N-glycosylated in an approximate 1 : 1 ratio, far less than that observed when NKCC1 was overexpressed in mammalian cells [16, 21]. These differences in NKCC1 behavior when endogenously expressed or when its expression is forced in cell lines are not out of the ordinary; enzymatic N-glycan biosynthesis depends on the level and transit of substrate proteins* en route* to their destination [45]. Therefore, it is expected that the proportion of complex N-glycosylated NKCC1 expressed at a given time may be variable and dependent upon the precursor protein reaching the appropriate N-glycosylation machinery to reach a given degree of N-glycan elaboration and decoration to be considered complex N-glycosylated, a task easily achieved when NKCCs are overexpressed [16, 26].

The prevailing consensus indicates that complex N-glycosylation is necessary for NKCCs to leave Golgi cisternae for plasma membrane delivery [16, 21, 64]. Among the many molecular signals potentially involved in plasma membrane targeting of NKCC1, N-glycosylation has indeed been assumed to be one of them. Our experiments revealed the presence of low levels of potentially complex N-glycosylated NKCC1 (~170 kDa) at the plasma membrane and much higher levels of core/high-mannose NKCC1 (~130 kDa) (Figure 4). In fact, ~10% of total NKCC1 was roughly estimated at the plasma membrane under basal conditions (Figures 4(a)–4(c)), mostly of the core/high-mannose N-glycan type. Although the proportion of total NKCC1 in the plasma membrane estimated in our experiments is well within the ranges recently reported for other transporters including the Na^+^/H^+^ exchanger-3 (NHE3) [65], several members of the* Slc26a* family of transporters [66], or channels such as CFTR [67], the possibility of contamination with intracellular NKCC1 as the source of ~130 kDa core/high-mannose N-glycosylated transporters in the plasma membrane may still remain. However, GAPDH, an abundant cytoplasmic protein expressed in all cells, was minimally detected, if any, in biotinylated preparations (see Figures 4(a), 8(c), 9(c), and 9(f)). Furthermore, the presence of* “nonprocessed”* NKCC1 in the plasma membrane of cells should not be surprising when the concept is placed within the context of recently published evidence. For instance, core/high-mannose and hybrid/complex-type* Slc12a5* (KCC2) and* Slc12a6* (KCC3) do reach the plasma membrane of oocytes overexpressing these proteins [68] and both 130 kDa and 170 kDa NKCC1 have been detected in membrane fractions of spinal cord neurons [69]. In addition,* Slc26a1* is found in the plasma membrane as complex and high-mannose N-glycans, whereas* Slc26a4* traffics to the plasma membrane fully deglycosylated [66], in analogy to* Slc4a1*, which is also found at the plasma membrane fully deglycosylated [70]. Therefore, when taken together, our results are compatible with the hypothesis that plasma membrane location of NKCC1 does not depend on the N-glycan nature of the transporter and, therefore, NKCC1 plasma membrane location may reflect its biosynthetic levels.

As any protein with predicted transmembrane domains, NKCC1 is intuitively expected to be present, to some extent, in membrane compartments including ER, Golgi cisternae, endosomes, and lysosomes. Except for the latter, where we could not find clear immunological evidence of NKCC1 localization (*data not shown*), the transporter was found in CRT-, Rab11-, and Man2-positive compartments (Figures 5–7). Further, approximately half of endogenous NKCC1 localized in the ER (Figure 5), a finding in line with the observation that ~50% of endogenous NKCC1 is core/high-mannose N-glycosylated (Figures 3(c) and 3(d)) and with the fact that core/high-mannose N-glycan proteins are invariably biosynthesized in the ER [49]. However, not all immunoreactive NKCC1 was found in ER. In fact, depleting NKCC1 from ER with cycloheximide resulted in the dispersion of NKCC1 into nonreticular compartments morphologically assessed as small vesicles suffused throughout the cytoplasm or condensed as rings around nuclei (Figures 5(f)–5(i)). This fine vesicular distribution of NKCC1 in COS7 cells is typical of the recycling endosome compartment [47]. In line, NKCC1 and Rab11, an endosome marker, colocalized (Figure 6). Furthermore, since complex N-glycosylation of proteins commences in the medial-Golgi, it follows that some proportion of total endogenous NKCC1 must be found in this organelle. Our results suggest colocalization of endogenous NKCC1 and Man2 (Figure 7), a* cis*/medial-Golgi resident enzyme [56]. However, whether this step is necessary for plasma membrane targeting of the transporter is debatable. Our results showed that TUN, a potent inhibitor of the first step of N-glycan biosynthesis, significantly reduced total NKCC1 protein levels (Figure 8), supporting the hypothesis that core N-glycosylation is important for NKCC1 biosynthesis and therefore trafficking. Indeed, the TUN-mediated decrease in total NKCC1 expression paralleled the reduced levels of NKCC1 expression at the plasma membrane (Figures 8(c) and 8(d)), a finding compatible with the notion that plasma membrane availability of the transporter depends on its biosynthetic levels (Figure 8(e)) rather than its N-glycan state, because proteins that cannot be N-glycosylated cannot mature and are frequently degraded. Therefore, the role of N-glycan maturation on plasma membrane targeting cannot be directly addressed by using TUN. Accordngly, we used KIF, an ER-mannosidase-I inhibitor, which precludes N-glycan maturation without impacting protein stability. Our results demonstrated that KIF increased total expression of EndoH-sensitive, core/high-mannose, and hybrid-type N-glycosylated NKCC1 (Figures 9(a), 9(b), and 9(g)), as expected for impaired mannose trimming in the ER [52, 54]. Interestingly, KIF increased the levels of hybrid-type N-glycan NKCC1 in the plasma membrane (Figures 9(c) and 9(h)) supporting the notion that NKCC1 of any N-glycan nature may reach that compartment. However, complex N-glycosylation also depends on demannosylated N-glycan substrates in the medial-Golgi. Therefore, KIF cannot exclude the possibility of a potential involvement of complex N-glycosylation in plasma membrane sorting. We addressed this issue by blocking Man2, the enzyme responsible of the first step of complex N-glycan biosynthesis. Our results demonstrate that SWN eliminated NKCC1 complex N-glycosylation and increased total and plasma membrane located core/high-mannose and hybrid-type N-glycosylated NKCC1 (Figures 9(d)–9(h)) suggesting that NKCC1 expression levels (of any N-glycan nature) parallel the levels of transporter in the plasma membrane and, therefore, plasma membrane located NKCC1 reflects its biosynthesis.

It has been suggested that N-glycosylation of membrane transporters of the* Slc12a* family plays an important role in their functional activity [23–26, 71, 72]. Interestingly, site-directed mutagenesis of the two N-glycan sequons present in rat* Slc12a3* almost abolished NCC-mediated cotransport without eliminating plasma membrane expression in* X. laevis* oocytes [71] whereas elimination of N-glycan sequons in the flounder* Slc12a3* or rat* Slc12a1* reduced both surface expression and total transport activity by ~50% [22, 73] suggesting that N-glycosylation is important for either plasma membrane targeting, cotransport function, or both. Our results suggest that COS7 cells upload Cl^−^ in a BTD-sensitive manner (Figure 9(i)) indicating that plasma membrane located NKCC1, either core/high-mannose, hybrid-, complex-type N-glycosylated, or a combination, thereof does transport Cl^−^. These findings support at least three hypotheses: (i) NKCC1 function is represented by the core/high-mannose type, the most abundant form of the transporter in the plasma membrane of COS7 cells (Figure 4(a)), (ii) all N-glycan species of NKCC1, that is, core/hybrid- and complex-type, are active, and (iii) core/high-mannose/hybrid NKCC1 are transport-deficient and, therefore, complex N-glycosylated NKCC1 represents the functional pool of the transporter. Our results support the latter case because treatment of COS7 cells with KIF or SWN, which eliminated complex N-glycosylated NKCC1 from the plasma membrane, also abolished BTD-dependent Cl^−^ uptake (Figure 9(j)), suggesting that core/high-mannose-type NKCC1 does not support Cl^−^ uptake despite its presence in the plasma membrane relative to complex N-glycosylated NKCC1, which may represent the active form of the transporter.

## 5. Conclusions

We have presented evidence for a key role of complex N-glycosylation in the cotransport function of NKCC1 but not for its targeting to the plasma membrane. In fact, the degree of NKCC1 targeting to this compartment may depend on its biosynthetic levels rather than the degree of attainable complex N-glycosylation. These conclusions, together with the fact that endogenous NKCC1 is also present in intracellular compartments of many cell types, have potential ramifications, particularly within functional interpretation of results, as most of plasma membrane located NKCC1 appears unable to upload Cl^−^ into the cell whereas a small proportion of plasma membrane NKCC1, which is complex N-glycosylated, may represent the functionally active pool of the transporter. Further, our results raise the possibility that intracellular Cl^−^ regulation and the cellular functions that depend on [Cl^−^]_i_ (*e.g.*, GABAergic signaling, cell volume, neuropeptide, or hormone release) may be sensitive to ER stress or nutrient availability. In addition, it remains open the possibility that core/hybrid-type NKCC1 located in the plasma membrane may have a role in cell physiology independently of its transport function.
